# Genome-wide analysis of the *FBA* gene family in soybean and their expression profile under salt-alkali stress

**DOI:** 10.3389/fpls.2026.1908014

**Published:** 2026-09-10

**Authors:** Xinyan Zhao, Yawen Zhan, Shuangjin Fan, Le Wang, Chenxi Zang, Yongwang Sun

**Affiliations:** College of Agriculture and Biology, Liaocheng University, Liaocheng, China

**Keywords:** abiotic stress and biotic stress, fructose-1,6-bisphosphate aldolase, genome-wide analysis, salt-alkali stress, soybean, transcriptome

## Abstract

Fructose-1,6-bisphosphate aldolases (FBAs; EC 4.1.2.13) are core enzymes involved in glycolysis, gluconeogenesis, and the Calvin cycle, exerting vital functions in carbon allocation, plant growth and development, as well as the adaptation to abiotic stresses. Although the *FBA* gene family has been systematically characterized in a variety of plant species, a comprehensive characterization of FBAs in soybean (*Glycine max*) remains unreported to date, restricting the elucidation of the conserved and legume-specific functions of *GmFBA* genes in stress adaptation. In the present study, a total of 13 *FBA* genes, designated as *GmFBA1* to *GmFBA13*, were identified in the soybean genome, which were unevenly distributed across 9 out of the 20 soybean chromosomes. Subcellular localization prediction showed that six GmFBA proteins were localized in the chloroplast, while the remaining seven were distributed in the cytoplasm. Phylogenetic analysis classified these genes into four distinct classes, and members within the same class shared similar gene structural features. Synteny analysis demonstrated that segmental duplication events served as a major driving force for the expansion of the *GmFBA* gene family. Promoter sequence analysis revealed that the upstream regulatory regions of *GmFBA* genes contained a variety of *cis*-acting elements associated with hormone responses and stress tolerance, suggesting that the expression of *GmFBA* genes might be modulated by diverse developmental cues and environmental stresses. Expression profiling indicated that *GmFBA* genes displayed differential expression patterns in various organs and tissues of soybean, and a number of *GmFBA* genes showed significant expression changes under biotic and abiotic stress conditions. Transcriptome analysis revealed distinct expression patterns of *GmFBA* genes in response to salt-alkali stress. Furthermore, quantitative real-time PCR assays indicated a significant upregulation of two specific *GmFBA* genes, *GmFBA2* and *GmFBA11*. These findings suggest that *GmFBA2* and *GmFBA11* may serve as candidate genes potentially involved in the adaptive response to salt-alkali stress. Collectively, this study presents the first comprehensive characterization of the *FBA* gene family in soybean, puts forward putative candidate genes for salt-alkali tolerance breeding, and lays a foundation for subsequent functional investigations.

## Introduction

Fructose-1,6-bisphosphate aldolase (FBAs; EC 4.1.2.13) catalyzes the reversible aldol condensation reaction between dihydroxyacetone phosphate (DHAP) and glyceraldehyde-3-phosphate (GAP) to produce fructose-1,6-bisphosphate (FBP), thus playing a central role in carbon metabolism and intracellular energy homeostasis (Berg et al., 2010; Fushinobu et al., 2011). This catalytic process serves as a fundamental step in several key metabolic pathways, including glycolysis, gluconeogenesis, and the Calvin-Benson cycle (Zgiby et al., 2000; Capodagli et al., 2014; Carrera et al., 2021).

Based on their catalytic characteristics and evolutionary origin, FBAs are generally divided into two distinct classes, which differ significantly in terms of structure and function (Nakahara et al., 2003). Class-I FBAs usually exist as homotetrameric proteins and form a covalent Schiff base intermediate with their substrates during the catalytic process. These enzymes utilize a conserved lysine residue to generate a nucleophilic enamine from DHAP, and their catalytic activity is not affected by ethylenediaminetetraacetic acid (EDTA) or regulated by potassium ions (Gross et al., 1999). In contrast, Class-II FBAs are homodimeric and require divalent metal cations as essential cofactors to stabilize the DHAP enolate intermediate, which is critical for the aldol condensation reaction, making them sensitive to EDTA inhibition (Gross et al., 1999). Class-I FBAs are distributed in a subset of bacterial species, as well as in animals and plants, while Class-II FBAs are mainly present in most bacteria, yeasts, fungi, and some higher plants (Penhoet et al., 1967; Tolan et al., 1987; Henze et al., 1998). In higher plants, FBA isoforms are localized in two different subcellular compartments: the cytoplasm (cFBA) and chloroplasts/plastids (cpFBA) based on their subcellular locations, where they play distinct roles in glycolysis and the Calvin cycle, respectively (Lebherz et al., 1984; Carrera et al., 2021). Both cFBA and cpFBA are encoded by independent nuclear genes, which are speculated to have originated from the duplication of a common ancestral gene (Anderson et al., 1995).

Gene family analysis is regarded as a fundamental approach for deciphering the function, structure, and evolutionary traits of genes (Su et al., 2022; Zhang et al., 2024a). To date, *FBA* gene family members have been identified in various plant species, with considerable variation in the number of these genes among different species. For example, five *FBA* genes have been identified in cucumber (*Cucumis sativus*) (Zhang et al., 2024b), seven in sweet potato (*Ipomoea batatas*) (Jiang et al., 2025), eight in *Arabidopsis thaliana*, tomato (*Solanum lycopersicum*), and rice (*Oryza sativa*) (Lu et al., 2012; Cai et al., 2016; Zhao et al., 2026), nine in maize (*Zea mays*), potato (*Solanum tuberosum*), and moso bamboo (*Phyllostachys edulis*) (Li et al., 2024b, c; Zhang et al., 2025), 16 in tobacco (*Nicotiana tabacum*) (Zhao et al., 2021), 17 in cotton (*Gossypium hirsutum*) (Li et al., 2021), 21 in wheat (*Triticum aestivum*) (Lv et al., 2017), and 22 in oilseed rape (*Brassica napus*) (Zhao et al., 2019). Despite the extensive characterization of the *FBA* gene family in various plant species, the evolutionary history and functional specialization of *FBA* genes in soybean remain poorly understood. Given that soybean has experienced two whole-genome duplication events (Schmutz et al., 2010), the *FBA* family may have undergone lineage-specific expansion and subfunctionalization that are distinct from those observed in *Arabidopsis* or rice.

Previous studies have confirmed that FBAs play a vital role in plant growth and development by regulating photosynthesis, carbon assimilation, and carbon partitioning. Changes in FBA activity have been demonstrated to influence photosynthesis and carbon allocation in potato (Haake et al., 1998). Overexpression of *AtFBA* was found to enhance the growth and photosynthetic capacity of tobacco plants (Uematsu et al., 2012). In the tea oil tree (*Camellia oleifera*), the expression levels of *CoFBA1* and *CoFBA3* are closely associated with tea tree oil content (Zeng et al., 2014). In rice, the expression of *OsAld-Y* was shown to participate in chlorophyll accumulation, chloroplast development, and plant growth by impacting the photosynthetic rate of leaves and rice sugar metabolism (Zhang et al., 2016). Reduced expression of *SlFBA7* resulted in smaller seed size and decreased biomass in transgenic tomato plants (Cai et al., 2018). Mutations in *AtFBA3* in Arabidopsis have been reported to impair plastid glycolytic metabolism in plant roots, thereby restricting the synthesis of essential compounds such as amino acids (Carrera et al., 2021). Additionally, numerous studies have indicated that *FBA* genes are involved in plant responses to various abiotic stresses, including salinity (Yamada et al., 2000; Fan et al., 2009), drought (Khanna et al., 2014; Oztur et al., 2002; Xue et al., 2008), and temperature stress (Michelis and Gepstein, 2000; Xue et al., 2008; Cai et al., 2018, 2022). Furthermore, *FBA* genes have been reported to participate in signal transduction (Oelze et al., 2014; Zhang et al., 2014), secondary metabolism (Zeng et al., 2014), and the regulation of plant responses to hormones (Konishi et al., 2004; Osakabe et al., 2005) and environmental light signals (Oelze et al., 2014). These findings suggest that *FBA* genes are potential targets for genetic engineering to improve crop yield, quality, and stress tolerance.

Soybean (*Glycine max*) is a major crop in global agriculture and serves as the primary source of plant-based protein, edible oil, and industrial raw materials (Coleman et al., 2021; Cao et al., 2024, 2025a, b). Numerous studies have reported the molecular mechanisms underlying soybean responses to various biotic and abiotic stresses (Yao et al., 2020; McCabe and Graham, 2020; DeMers et al., 2020; Tamang et al., 2021; Wang et al., 2023b). Among abiotic stresses, saline-alkali stress differs from single salt or alkali stress. It exerts composite damages including ion toxicity, osmotic imbalance and high-pH disturbance, which severely disrupt plant carbon-metabolic homeostasis and have become a major constraint limiting global crop production (Fan et al., 2012; Cao et al., 2023; Li et al., 2025a). Globally, approximately 40% of irrigated lands are affected by elevated soil salinity, and the progressive expansion of soil salinization and alkalization poses a major threat to crop performance and yield stability (Bailey-Serres et al., 2019; Chele et al., 2021). Genome-wide characterization of the evolution and stress-related functions of metabolic and regulatory gene families has become a cutting-edge strategy for mining stress-tolerance genes in soybean (Sui et al., 2023). For example, genome-wide identification of the *TPS* family has been conducted to profile its tissue-expression patterns (Li et al., 2024a); the cytokinin oxidase/dehydrogenase family was systematically characterized for its features and responses to diverse abiotic stresses (Du et al., 2023); the *AP2/ERF* family was comprehensively surveyed, and *GmAP2/ERF144* was functionally validated for drought tolerance (Wang et al., 2022). Nevertheless, as core components of carbon metabolism, the soybean *FBA* gene family has not been systematically elucidated, representing an important research gap in this field. Therefore, we performed genome-wide identification and systematic analysis of the soybean *FBA* gene family, and characterized its genomic structures, tissue-specific expression profiles, as well as its expression patterns under saline-alkali stress. This study aims to dissect the potential biological functions of *GmFBA* genes and provide fundamental data as well as candidate gene resources for mechanistic investigation and molecular breeding toward improved saline-alkali tolerance in soybean.

## Materials and methods

### Identification of *FBA* genes in the soybean genome

To identify *FBA* family members in the soybean genome (Wm82.a4.v1), two complementary approaches were employed: Hidden Markov Model-based searches (http://hmmer.org/) and BLASTP (https://blast.ncbi.nlm.nih.gov/Blast.cgi). Soybean genome sequences, protein sequences, CDS sequences, and GFF3 annotation files were acquired from the Phytozome database (https://phytozome-next.jgi.doe.gov/). The HMM profile corresponding to FBA proteins (PF00274) was retrieved from the Pfam database (http://pfam.xfam.org/). In addition, eight FBA protein sequences from *Arabidopsis* were obtained from the TAIR database (Lu et al., 2012; https://www.arabidopsis.org/) and used as queries in BLASTP searches against the soybean proteome. The candidate sequences obtained from HMMER and BLASTP were merged, and redundant entries were removed. The remaining non-redundant sequences were further verified using the InterPro database (https://www.ebi.ac.uk/interpro/) to confirm the presence of the Glycolytic domain. Only sequences harboring a complete Glycolytic domain were designated as bona fide soybean *FBA* family members, which were subsequently named based on their physical positions on the chromosomes.

### Prediction of the physicochemical properties and subcellular localization of GmFBA proteins

The physicochemical characteristics of GmFBA proteins, including molecular weight, isoelectric point, instability index, aliphatic index and grand average of hydropathicity (GRAVY), were analyzed using the ProtParam tool available on the ExPASy server (https://web.expasy.org/protparam/). Meanwhile, the subcellular localization of each GmFBA protein was predicted with the Plant-mPLoc webtool (Chou and Shen, 2010; http://www.csbio.sjtu.edu.cn/bioinf/plant-multi/).

### Chromosomal localization and synteny analysis of *GmFBA* genes

Chromosomal locations of the *GmFBA* genes were retrieved from the corresponding soybean genome annotation files. For synteny analysis, an all-against-all BLASTP search was performed across the soybean genome, with an E-value threshold set at 10-10, to identify gene pairs. The resulting BLASTP outputs were then imported into the MCScanX toolkit embedded in TBtools (Chen et al., 2020) to detect syntenic blocks and characterize gene duplication events. A circular diagram illustrating collinear *GmFBA* gene pairs was generated using the Advanced Circos plugin in TBtools, where duplicated gene pairs are linked by solid red lines. The non-synonymous substitution rate (*K*a) and synonymous substitution rate (*K*s) of these duplicated pairs were calculated via the *K*a*/K*s Calculator module in TBtools, to assess the evolutionary divergence of the paralogous genes. The duplication time was calculated according to published method by using the following formula: Time = *K*s/(2 × substitution rate) and the substitution rates of soybean is 6.1 × 10–^9^ site per year (Fan et al., 2013).

### Phylogenetic analysis of FBA proteins

Multiple sequence alignment and phylogenetic analysis of FBA proteins were conducted using MEGA 11.0 software (https://www.megasoftware.net/), involving protein sequences from four plant species: *Arabidopsis* (Lu et al., 2012), soybean, rice (Zhao et al., 2026), and tomato (Cai et al., 2016). Following sequence alignment, a neighbor-joining (NJ) phylogenetic tree was constructed with the Poisson model, using 1000 bootstrap replicates and the pairwise deletion parameter to ensure the reliability of the tree topology.

### Analysis of gene structure, *cis*-acting elements and conserved motif of *GmFBA* genes

The nucleotide sequence structure of *GmFBA* genes was analyzed using the soybean GFF3 annotation file, and the exon-intron distribution map was visualized with TBtools software. For promoter analysis, a 2.0 kb sequence upstream of each *GmFBA* gene was extracted from the soybean genome as the promoter region. The PlantCARE website (https://bioinformatics.psb.ugent.be/webtools/plantcare/html/) was employed to predict cis-acting elements within these promoter regions, which were further classified into hormone-responsive and stress-responsive elements based on their putative sequences and functions. Additionally, conserved motifs of GmFBA proteins were identified via the MEME online tool (http://meme-suite.org/) with the parameter settings as follows: a maximum of 10 motifs and an optimal motif width ranging from 6 to 200 amino acid residues. The visualization of the identified conserved motifs was implemented using TBtools software.

### Expression pattern of *GmFBA* genes in various organs/tissues

RNA-seq data from seven tissues/organs of soybean were downloaded from the SoyOmics database (https://ngdc.cncb.ac.cn/soyomics/index), including the roots, cotyledons, stems, leaves, flowers, and developing pods and seeds. The expression levels of each gene were represented by its fragments per kilobase of exon per million fragments mapped (FPKM) values, and a heatmap showing tissues-specific expression profiles was generated using the log_2_-transformed (FPKM + 1) values of *GmFBA* genes in the TBtools software. For stress-responsive expression analysis, RNA-seq data under multiple biotic (7 conditions: aphid infestation, Phialophora gregata, *Aspergillus oryzae*, *Rhizopus oligosporus*, soybean mosaic virus, *Fusarium virgiforme*, and cyst nematode) and abiotic (7 conditions: drought, submergence, dehydration, cold, heat, drought and heat, and salt) stress experiments were acquired from the Plant Public RNA-seq Database (Yu et al., 2022; https://www.plantrnadb.com/), with experimental details of stress treatments provided in **Supplementary Table 4**. Only pre-computed FPKM values were retrieved from this database. Fold-change values were calculated between stress-treated and control samples. Genes with a fold-change ≥ 2.0 were defined as upregulated, whereas genes with a fold-change ≤ 0.5 were considered downregulated.

### Plant growth, salt-alkali stress treatments of soybean and transcriptome

The soybean cultivar “QH34” was used in this study. Its salt-tolerant phenotype has been well-documented in previous research (Hu et al., 2022). This tolerant genotype was selected to facilitate the identification of stress-responsive candidate genes. Seeds were sown in black plastic culture boxes filled with vermiculite. Plants were grown in a controlled environment chamber under a 16 h light (26 °C)/8 h dark (22 °C) photoperiod. At the V_1_ stage (approximately 14 days after sowing; Fehr and Caviness, 1977), uniformly sized seedlings were selected for salt-alkali stress treatment. Salt-alkali stress was applied using a hydroponic system. A stock solution of mixed salts was prepared containing NaCl, Na_2_CO_3_, NaHCO_3_, and Na_2_SO_4_ at a molar ratio of 1:1:9:9 (Cao et al., 2023), which was diluted 10-fold with 1/2 Hoagland nutrient solution to obtain the final treatment solution containing 150 mM Na^+^ at pH 8.9. Seedlings were transferred to either control (0 mM Na^+^, 1/2 Hoagland solution) or salt-alkali treatment (150 mM Na^+^, pH 8.9) solutions, with three biological replicates (six seedlings per replicate) for each treatment. Root tissues were collected at 2 h, 6 h, 12 h, and 24 h after treatment initiation, frozen immediately in liquid nitrogen, and stored at −80 °C. For transcriptome sequencing, total RNA was extracted from the collected root samples using an RNA extraction kit (Li et al., 2025b). RNA integrity was verified using an Agilent 2100 Bioanalyzer. cDNA libraries were constructed and sequenced on the Illumina NovaSeq 6000 platform by Novogene (Beijing, China), generating 150 bp paired-end reads. Raw reads were processed using fastp (v1.0.1) (Chen et al., 2018; Xin et al., 2019) for quality control and mapped to the soybean reference genome (Wm82.a4.v1) using HISAT2 (v2.1.0) (Kim et al., 2019). Gene expression levels were quantified as FPKM using StringTie (v2.2.3) (Pertea et al., 2015), and differential expression analysis between treatment and control groups was performed using DESeq2 (v1.42.0) (Love et al., 2014), with |log_2_FC| ≥ 1 and FDR < 0.05 as the threshold for significance. The raw sequencing data have been deposited in the NCBI Sequence Read Archive (SRA) under BioProject accession number PRJNA1476727.

### RNA extraction, cDNA synthesis, and qRT-PCR analysis

Total RNA was isolated from soybean roots collected under control and salt-alkali stress conditions using a commercial plant RNA purification kit (TSINGKE, cat. TSP401, China), strictly following the recommended protocol (Cheng et al., 2024). In brief, root samples were homogenized to a fine powder in liquid nitrogen, and approximately 100 mg of the powdered tissue was mixed with 0.45 µl of Buffer RL. The RNA was captured and purified using RNase-Free Columns CS and finally recovered in 30 µl of RNase-free ddH_2_O. First-strand cDNA was synthesized from 1 µg of total RNA using a reverse transcription kit (TSINGKE, cat. TSK301S, China), and the resulting cDNA was kept at −20 °C until further use. For qRT-PCR, each reaction was prepared using a SYBR Green master mix (TSINGKE, cat. TSE201, China) and run on a LightCycler 480 system (Roche). The thermal cycling program consisted of an initial denaturation at 95 °C for 30 s, followed by 40 cycles of 95 °C for 5 s and 60 °C for 30 s, with a melting curve analysis added at the end (Wang et al., 2023a). The soybean *GmTub* gene (*Glyma.05G157300*, Wang et al., 2016) was used as the internal reference for normalizing expression levels, which has been validated in our experimental conditions. Relative transcript abundances were calculated using the 2^-ΔΔCt^ method (Derveaux et al., 2010). All reactions were performed in three biological replicates, and results are presented as mean ± standard error (SE). Prior to statistical analysis, homogeneity of variances was assessed using the Brown-Forsythe test, and the data met this assumption. At each sampling time-point, gene-expression differences between the salt-alkali-treated group and its corresponding control group were analyzed using independent-samples *t*-tests. The significance thresholds were set at *p* < 0.05 (*) or *p* < 0.01 (**). Data visualization was carried out with GraphPad Prism 8.0. Gene-specific primer sequences are provided in **Supplementary Table 1**.

## Results

### Identification of *GmFBA* genes and characterization of their protein physicochemical properties

**Figure 1:**
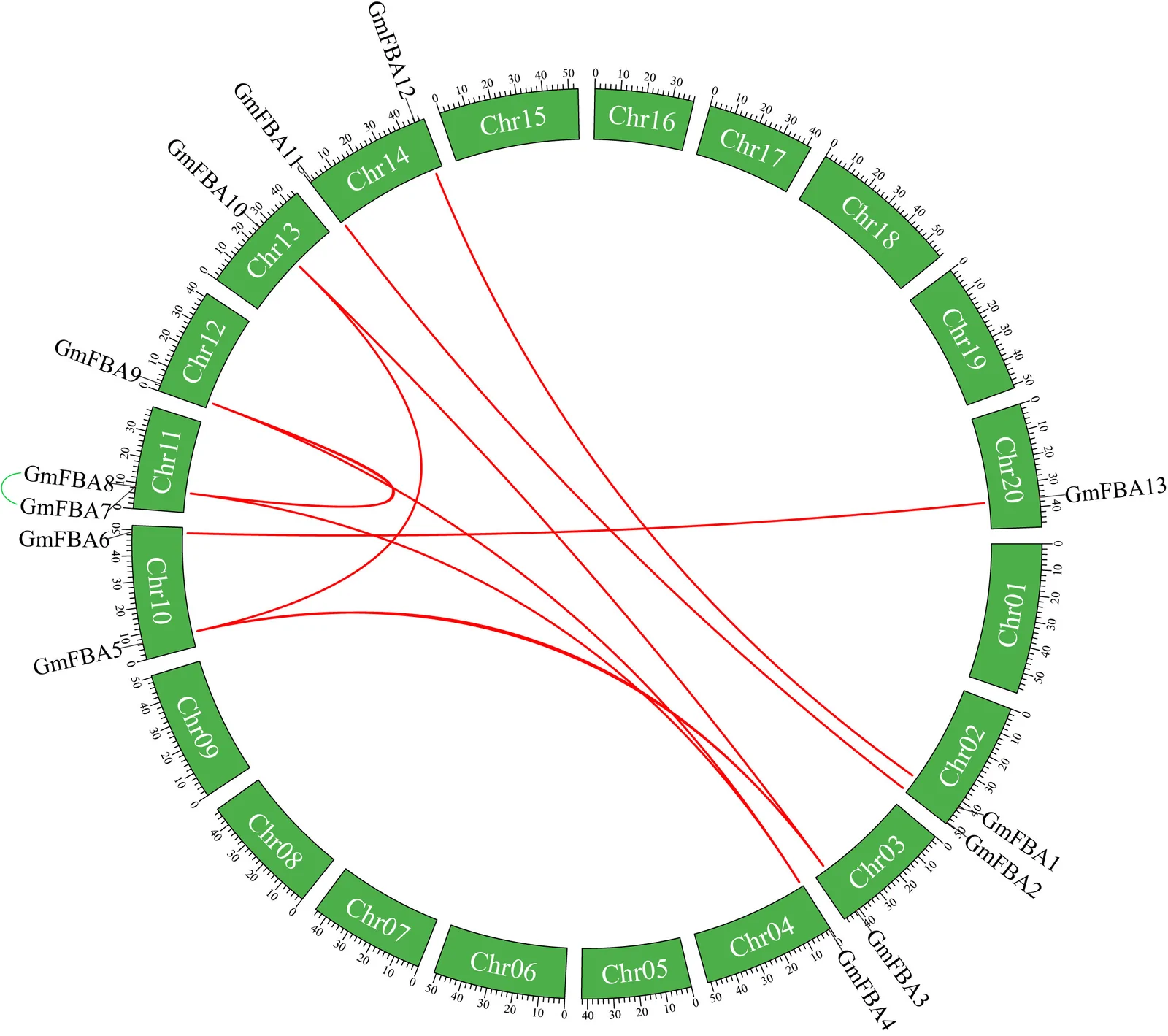
Chromosome location and syntenic relationships of *GmFBA* genes. The positions of *GmFBA* genes on soybean chromosomes are shown on the outer circle. Different boxes represent the 20 soybean chromosomes (Chr01-Chr20). Among them, *GmFBA7* and *GmFBA8* located on chromosome 11 correspond to a tandem-duplicated gene pair; all other connected gene-pairs are derived from segmental-duplication events. The segmental and tandem duplicated *GmFBA* genes are connected by red and green curves.

After integrating the search results of HMMER and BLASTP, 16 non-redundant proteins were obtained and subjected to conservative domain identification. A total of 13 proteins were found to contain the intact Glycolytic domain, and they were considered as *FBA* gene family members of soybean. The corresponding genes were designated as *GmFBA1* to *GmFBA13* according to their positions on soybean chromosomes (**Table 1**, **Figure 1**). The nucleotide sequence length of *GmFBA* genes varied from 1865 bp (*GmFBA4*) to 2979 bp (*GmFBA5*), and their protein length ranged from 357 (*GmFBA2* and *GmFBA11*) to 399 amino acids (*GmFBA7* and *GmFBA8*). Furthermore, the physicochemical properties of the GmFBA proteins were characterized. The molecular weight of GmFBA protein ranged from 38.26 kDa (GmFBA1) to 42.95 kDa (GmFBA7 and GmFBA8), and the theoretical isoelectric point ranged from 6.26 (GmFBA11) to 8.52 (GmFBA13), indicating that all GmFBA proteins tend to be neutral. Analysis of the instability index indicated that eleven proteins are potentially stable (< 40), while the remaining two are probably unstable proteins (> 40). The aliphatic index, which ranged from 86.10 (GmFBA6) to 92.96 (GmFBA12), indicated the thermal stability of GmFBA proteins. The GRAVY values of GmFBA proteins ranged from -0.236 (GmFBA6) to -0.097 (GmFBA4), suggesting that they are hydrophilic proteins. Subcellular localization prediction showed that GmFBA4, -6, -7, -8, -9, and -13 were localized in the chloroplast, and GmFBA1, -2, -3, -5, -10, -11, and -12 were localized in the cytoplasm.

**Table 1:** Basic information, physicochemical properties, and subcellular localization of GmFBAs.

Name	Gene ID	Chromosomal location	Genomic length (bp)	Protein length	Molecular weight (kDa)	Isoelectric point	Instability index	Aliphatic index	GRAVY	Subcellular localization
*GmFBA1*	*Glyma.02G222400*	Chr02:42831129-42834115	2441	358	38.26	7.12	34.52	92.68	-0.16	Cytoplasm
*GmFBA2*	*Glyma.02G303000*	Chr02:49670872-49673220	1929	357	38.38	6.78	34.98	92.13	-0.15	Cytoplasm
*GmFBA3*	*Glyma.03G191500*	Chr03:41397217-41400225	2553	358	38.51	6.35	30.89	86.73	-0.23	Cytoplasm
*GmFBA4*	*Glyma.04G008300*	Chr04:632970-635282	1865	395	42.57	6.38	32.93	92.91	-0.10	Chloroplast
*GmFBA5*	*Glyma.10G066700*	Chr10:6430040-6433728	2979	358	38.49	7.57	31.77	91.34	-0.21	Cytoplasm
*GmFBA6*	*Glyma.10G268500*	Chr10:49185548-49188294	2250	390	42.54	8.16	45.78	86.10	-0.24	Chloroplast
*GmFBA7*	*Glyma.11G111100*	Chr11:8476331-8479290	2490	399	42.95	8.24	31.99	90.53	-0.16	Chloroplast
*GmFBA8*	*Glyma.11G111400*	Chr11:8490115-8493641	2445	399	42.95	8.24	31.99	90.53	-0.16	Chloroplast
*GmFBA9*	*Glyma.12G037400*	Chr12:2702927-2705791	2444	398	42.87	6.86	32.29	89.77	-0.17	Chloroplast
*GmFBA10*	*Glyma.13G151800*	Chr13:25661266-25664559	2895	358	38.41	6.62	32.84	91.62	-0.20	Cytoplasm
*GmFBA11*	*Glyma.14G010900*	Chr14:825470-828183	1931	357	38.42	6.26	34.54	92.66	-0.15	Cytoplasm
*GmFBA12*	*Glyma.14G189400*	Chr14:46288834-46292019	2415	358	38.35	7.12	28.36	92.96	-0.17	Cytoplasm
*GmFBA13*	*Glyma.20G122500*	Chr20:36507646-36510422	2249	388	42.42	8.52	45.66	86.29	-0.26	Chloroplast

### Chromosomal distribution and duplication analysis of *GmFBA* genes

To determine the chromosomal distribution pattern of *GmFBA* genes, their physical positions across the soybean genome were examined. As shown in **Figure 1**, *GmFBA* genes were unevenly distributed across nine out of the twenty soybean chromosomes. Specifically, Chr02, Chr10, Chr11, and Chr14 each harbored two *GmFBA* genes, while Chr03, Chr04, Chr12, Chr13, and Chr20 each contained one *GmFBA* gene locus. It is widely recognized that segmental and tandem duplication events represent two primary evolutionary mechanisms responsible for the expansion of gene families across species (Lynch and Conery, 2000). To elucidate the driving forces under the expansion of the *GmFBA* gene family, a collinearity analysis was conducted. Our results identified nine pairs of segmentally duplicated genes and one pair of tandemly duplicated genes (*GmFBA7* and *GmFBA8*). To assess the selective pressure constraining the duplication of *GmFBA* genes, we calculated the ratios of *K*a to *K*s substitutions. The *K*a*/K*s values for the nine segmentally duplicated gene pairs ranged from 0.03 to 0.19 (**Table 2**). This consistent pattern of values far below 1 strongly suggests that *GmFBA* genes have evolved under the influence of strong purifying selection. Furthermore, it was estimated that the duplication events between *GmFBA* genes might occur at 5.84 to 203.04 million years ago (MYA) (**Table 2**).

**Table 2:** The *K*a/*K*s ratios of the duplicated *GmFBA* gene pairs.

Gene1	Gene2	*K*a	*K*s	*K*a/*K*s	Time (MYA)
*GmFBA1*	*GmFBA12*	0.0226	0.1175	0.1926	9.63
*GmFBA2*	*GmFBA11*	0.0087	0.0712	0.1226	5.84
*GmFBA3*	*GmFBA5*	0.0981	2.0110	0.0488	164.84
*GmFBA3*	*GmFBA10*	0.0899	2.4771	0.0363	203.04
*GmFBA4*	*GmFBA7*	0.0406	0.5004	0.0810	41.02
*GmFBA4*	*GmFBA9*	0.0453	0.4748	0.0955	38.92
*GmFBA5*	*GmFBA10*	0.0091	0.2523	0.0361	20.68
*GmFBA6*	*GmFBA13*	0.0034	0.1077	0.0319	8.83
*GmFBA7*	*GmFBA9*	0.0067	0.1151	0.0586	9.43

### Phylogenetic analysis of *GmFBA* genes

**Figure 2:**
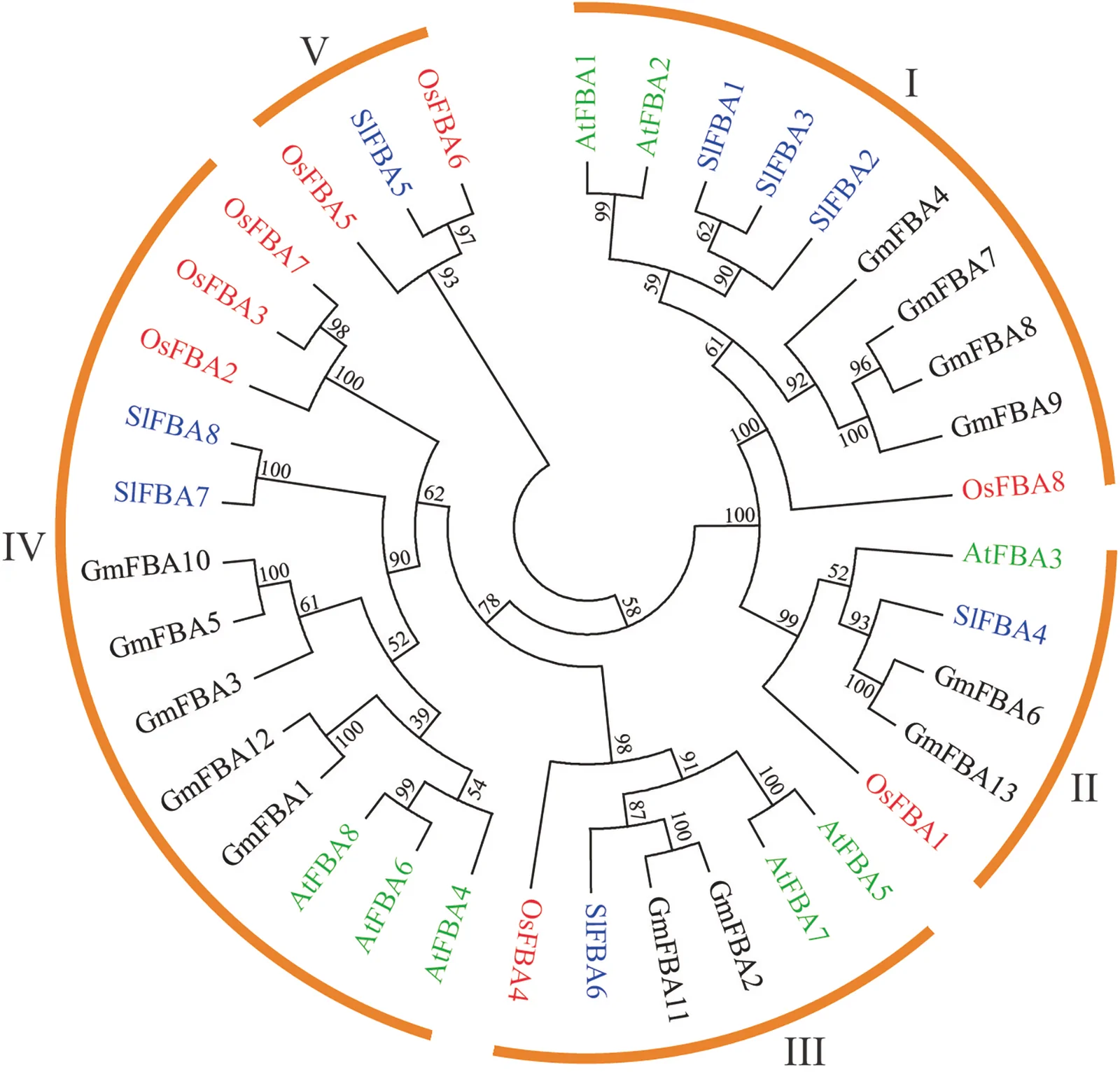
Phylogenetic relationship of FBA proteins from *Glycine max (Gm)*, *Arabidopsis thaliana (At), Oryza sativa (Os)*, and *Solanum lycopersicum (Sl)*. The tree was constructed using the neighbor-joining method with 1000 bootstrap replicates. The numbers next to the branch show the 1000 bootstrap replicates expressed in percentage.

To elucidate the evolutionary relationships of the *FBA* genes, a total of 37 FBA proteins from four species were used to construct a phylogenetic tree, including 8 each from Arabidopsis, rice, and tomato, and 13 from soybean. As shown in **Figure 2**, these FBAs were divided into five groups. Group V only contains two FBAs from rice and one FBA from tomato, while the other four groups contain FBAs from four species. Group IV contains the highest number (5) of GmFBAs, followed by group I (4), and groups II and III (2 each).

### Gene structure and motif composition of *GmFBAs*

**Figure 3:**
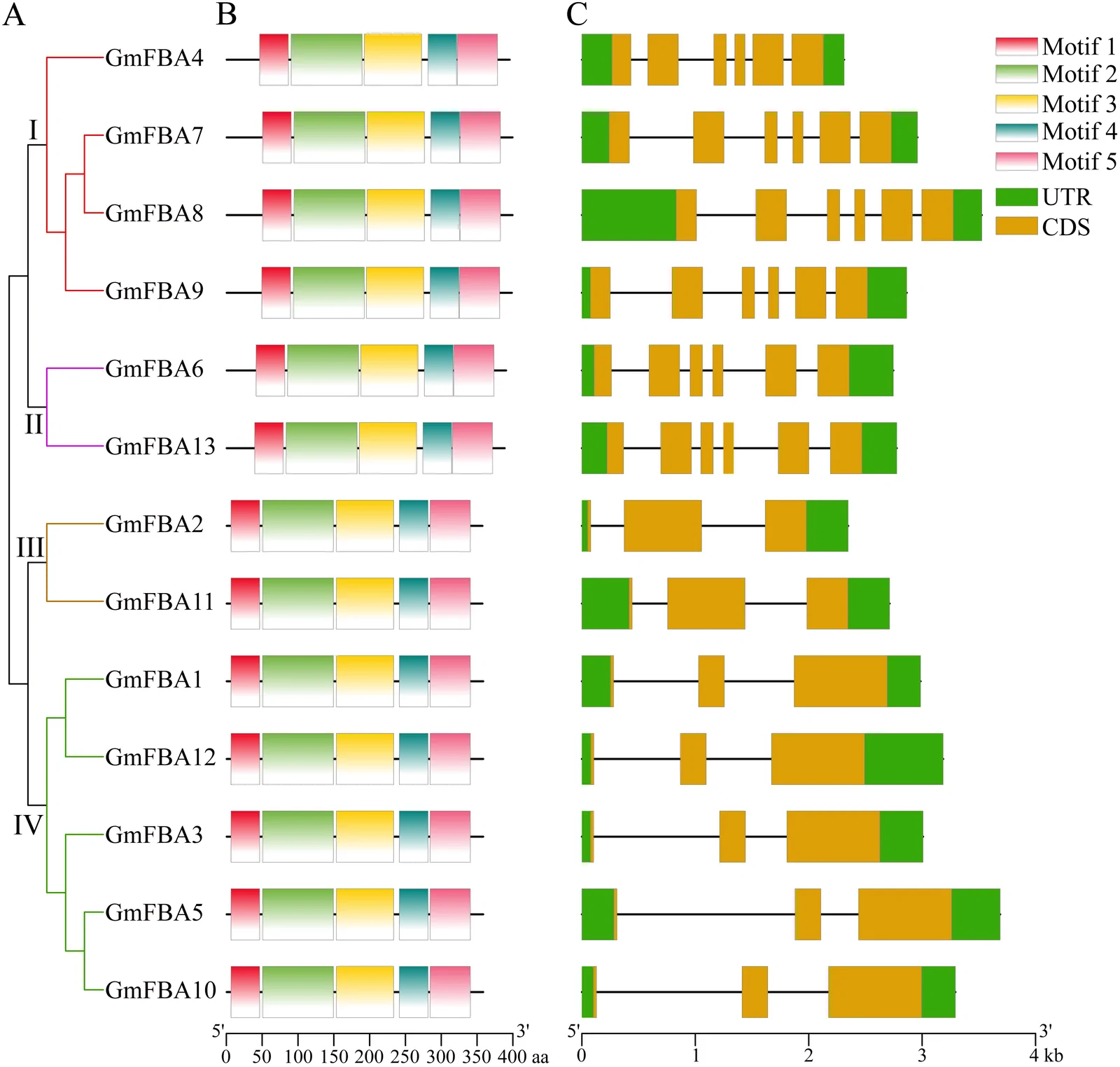
Schematic diagrams of *GmFBA* genes and motif composition of GmFBA proteins. **(A)** A rootless neighbor-joining tree was constructed based on the complete sequences of the 13 GmFBA proteins using MEGA 11.0 software. **(B)** Distribution map of conserved motifs in GmFBA proteins. The five putative motifs are represented by different colored boxes. The length of the exon-intron junctions and amino acid sequences is indicated by the ruler at the bottom. **(C)** Structural analysis of the exon-intron boundaries of the *GmFBA* genes, with yellow boxes indicating exons and black lines indicating introns.

To investigate the structural composition of *GmFBA* genes, their exon-intron organizations were determined by aligning the CDS of each *GmFBA* gene with its corresponding genomic DNA sequence (**Figure 3**). Detailed exon-length information for each *GmFBA* gene was provided in **Supplementary Table S3**. The results indicated that *GmFBA* genes belonging to the same phylogenetic group exhibited analogous gene structural patterns. Specifically, all *GmFBA* members in groups I and II contained six exons separated by five introns, whereas those in groups III and IV consisted of three exons and two introns. Motif analysis was further performed to explore the conserved characteristics of GmFBA proteins, and a total of five conserved motifs were identified across all *GmFBA* sequences (**Supplementary Figure 1**). Notably, all GmFBA proteins shared an identical motif distribution profile. In addition, compared with GmFBA proteins in groups III and IV, those in groups I and II possessed a longer N-terminal extension (34–44 amino acids). Subcellular localization prediction revealed that GmFBA proteins in groups I and II were predicted to be localized in the chloroplast, while those in groups III and IV were predicted to be cytoplasmic. These findings suggest that the longer N-terminal extension in GmFBA proteins of groups I and II may be associated with their chloroplast targeting and localization.

### Distribution of *cis*-acting elements in *GmFBA* gene promoters

**Figure 4:**
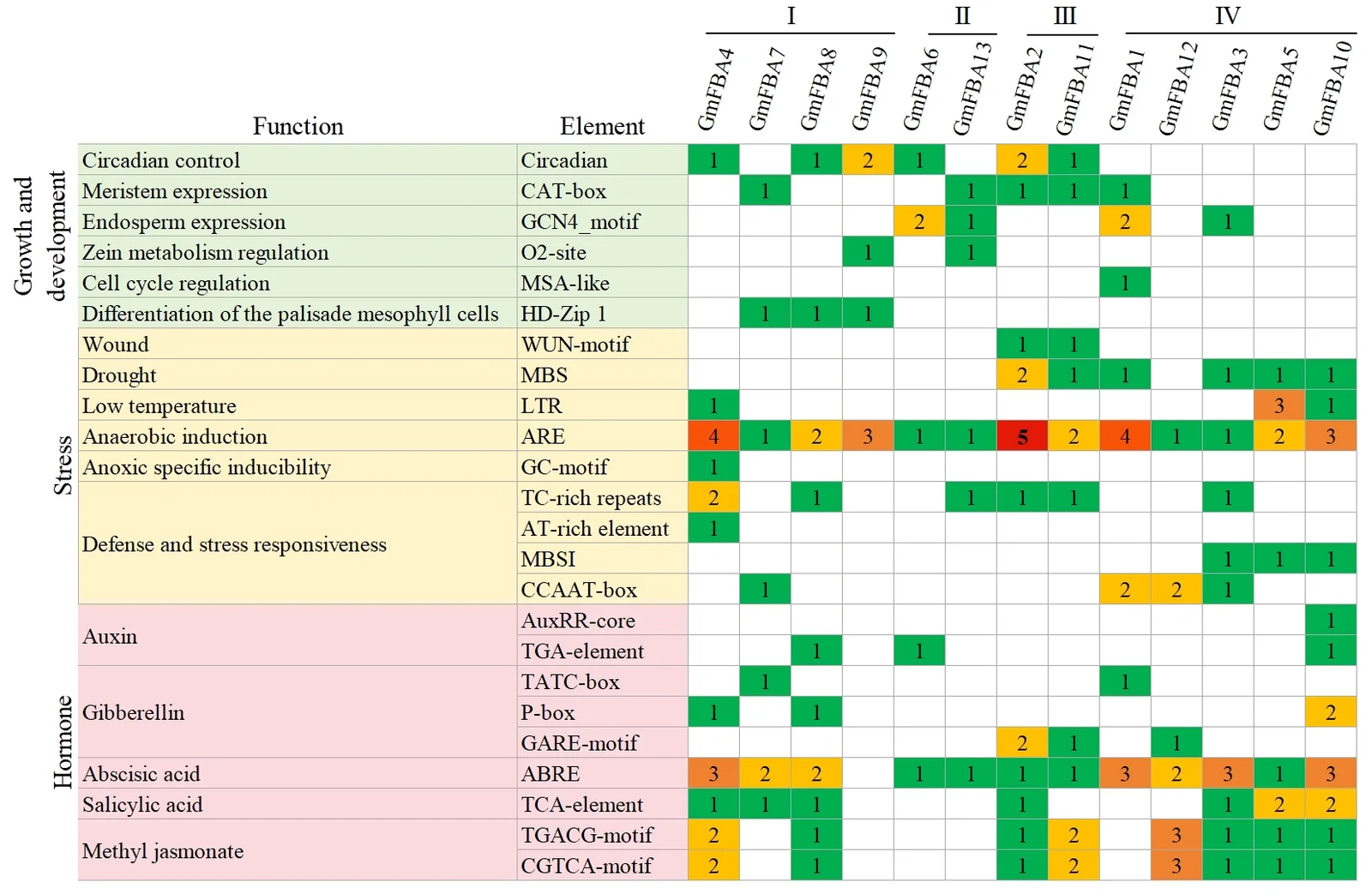
*Cis*-acting elements in the promoter regions of the 13 *GmFBA* genes. The color and number of the grid indicate numbers of different *cis*-acting elements in *GmFBA* genes.

To explore the regulatory signals that affect gene expression, a 2.0 kb sequence upstream of the start codon of each *GmFBA* gene was retrieved and uploaded to the PlantCARE website. A total of 24 *cis*-acting elements associated with growth and development, stress responses, and hormone regulation were identified (**Figure 4**). Among the *cis*-acting elements related to growth and development, the most abundant ones were those involved in circadian control (8), endosperm expression (6), meristem expression (5), palisade mesophyll cell differentiation (3), zein metabolism regulation (2), and cell cycle regulation (1). It is worth noting that *GmFBA1, GmFBA9*, and *GmFBA13* each contained three different types of growth- and development-associated *cis*-acting elements. With respect to stress-responsive *cis*-acting elements, anaerobic induction elements were the most prominent (30), followed by elements related to defense and stress responsiveness (17), drought responsiveness (7), low-temperature responsiveness (5), wound responsiveness (2), and anoxic specific inducibility (1). These findings imply that the *GmFBA* gene family might exert a crucial regulatory function in soybean’s adaptation to various abiotic stresses. Notably, anaerobic induction elements were detected in all *GmFBA* genes, with *GmFBA13* carrying five copies of this element. In addition, five distinct classes of hormone-responsive *cis*-acting elements were identified in the promoters of *GmFBA* genes. The most prevalent hormone-responsive elements were those responsive to methyl jasmonate (27), followed by abscisic acid (23), gibberellin (10), salicylic acid (9), and auxin (4). Specifically, *GmFBA8* and *GmFBA10* were found to contain all five types of hormone-responsive *cis*-acting elements.

### Expression profiles of *GmFBA* genes in different tissues of soybean

**Figure 5:**
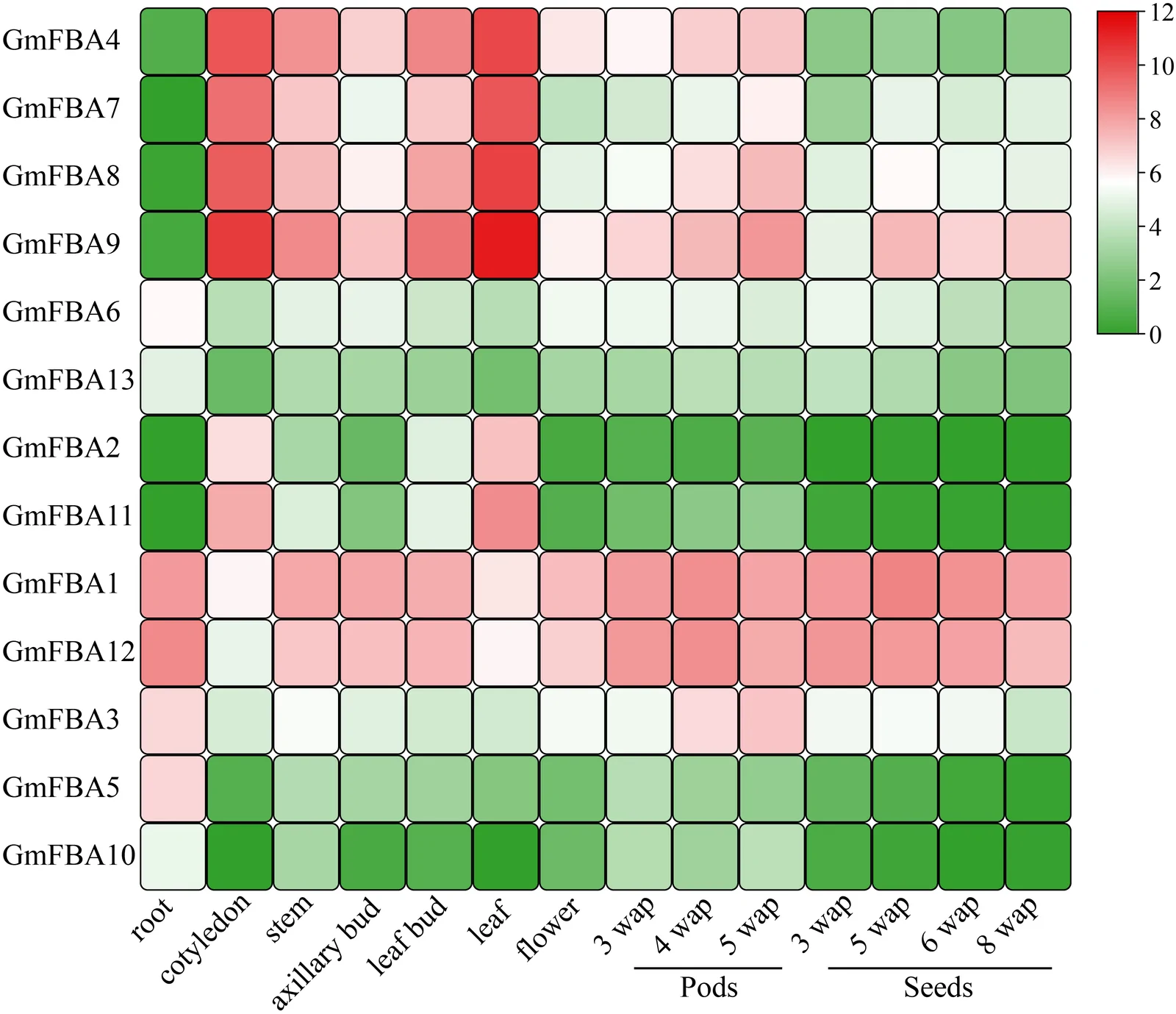
Expression profiles of the *GmFBA* genes. The transcription levels of *GmFBA* genes in nine selected tissues or organs of soybean plants were analyzed based on data collected from SoyOmics. Tissues and organs analyzed include roots, cotyledons, stems, axillary buds, leaf buds, leaves, flowers, and developing pods and seeds at 3, 4, 5, 6, and 8 weeks after pollination (wap). A heatmap was generated using TBtools. The color scale from green to red indicates the increased expression levels of the genes.

To investigate the spatiotemporal expression characteristics of *GmFBA* genes, we analyzed RNA-seq transcriptomic datasets retrieved from the SoyOmics database. These datasets cover seven vegetative and reproductive organs (including roots, cotyledons, stems, axillary buds, leaf buds, leaves, and flowers) as well as developing pods and seeds at various post-flowering stages. The expression levels of target genes were quantified by FPKM. Genes with FPKM values below 1.00 were defined as non-expressed, while those with FPKM values ranging from 1.00 to 20.00 were considered lowly expressed, and genes with FPKM ≥ 20.00 were classified as highly expressed. As illustrated in **Figure 5**; **Supplementary Table 2**, *GmFBA* genes showed distinct tissue- and stage-specific expression patterns. All *GmFBA* genes were expressed in at least one of the tested tissues or organs. Genes belonging to Group I were expressed in all organs except roots, with extremely high expression levels in vegetative organs such as cotyledons, stems, and leaves, suggesting that these genes play a crucial role in soybean growth and development. Group II genes exhibited moderate to high expression in most tissues and organs, with the highest expression observed in roots. Genes in Group III were mainly expressed in cotyledons, stems, leaf buds, and leaves, indicating their specific involvement in tissue-specific developmental processes and metabolic activities. In Group IV, *GmFBA1*, *GmFBA3*, and *GmFBA12* were highly and constitutively expressed across all tissues and organs, which implies their indispensable function in fundamental biological processes. In contrast, *GmFBA5* and *GmFBA10* displayed high expression in roots but low expression in aerial parts, suggesting that these two genes may be critical for root growth and development.

### Expression of *GmFBA* genes in response to biotic and abiotic stresses

**Figure 6:**
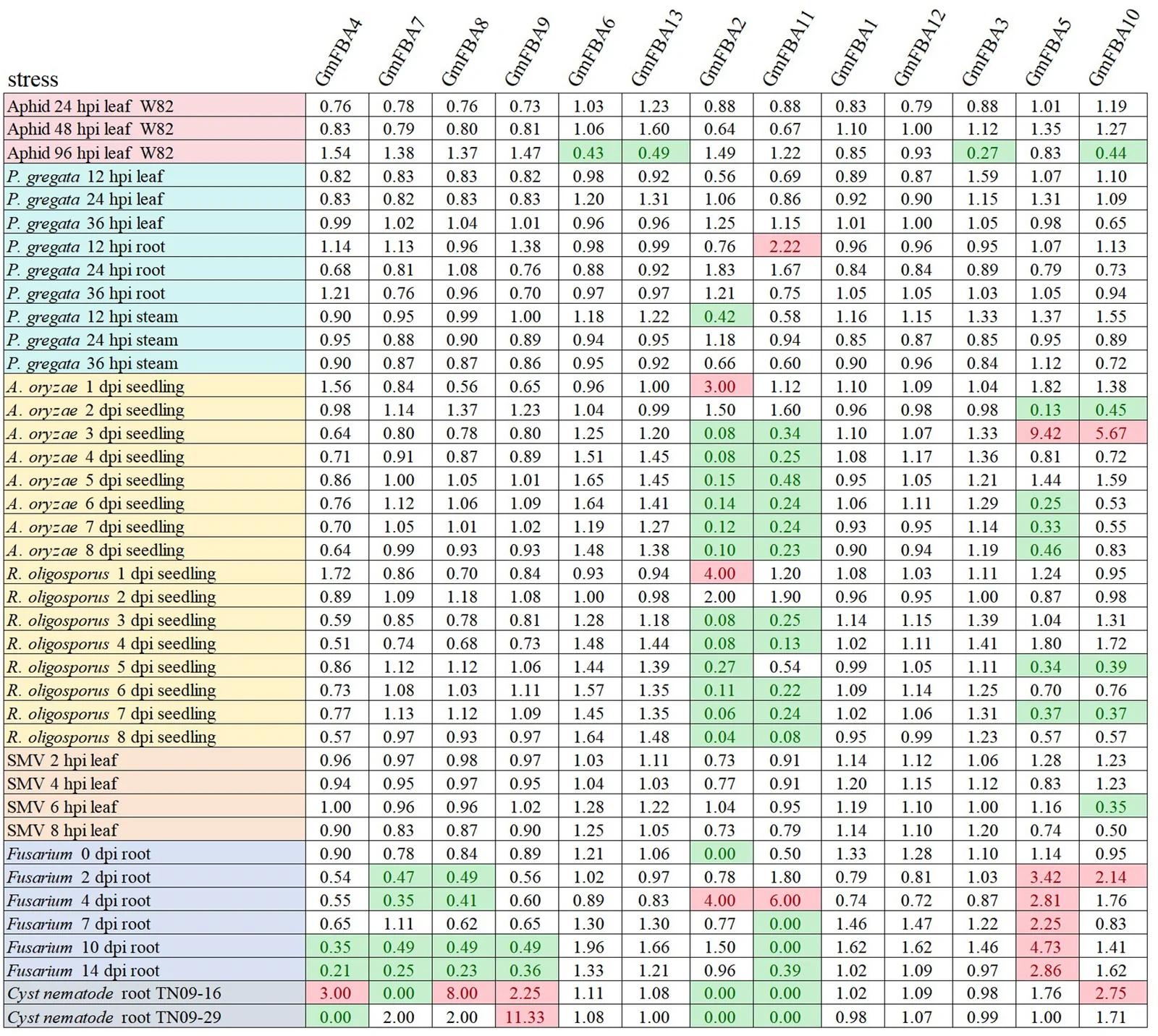
Expression level changes of *GmFBA* genes under various biotic stresses. Fold changes of expression level of *GmFBA* genes under multiple biotic stresses, including aphid infestation, *Phialophora gregata*, *Aspergillus oryzae*, *Rhizopus oligosporus*, soybean mosaic virus (SMV), *Fusarium virgiforme*, and cyst nematode. Fold changes higher than 2.0 or lower than 0.5 were highlighted by red and green colors, respectively.

**Figure 7:**
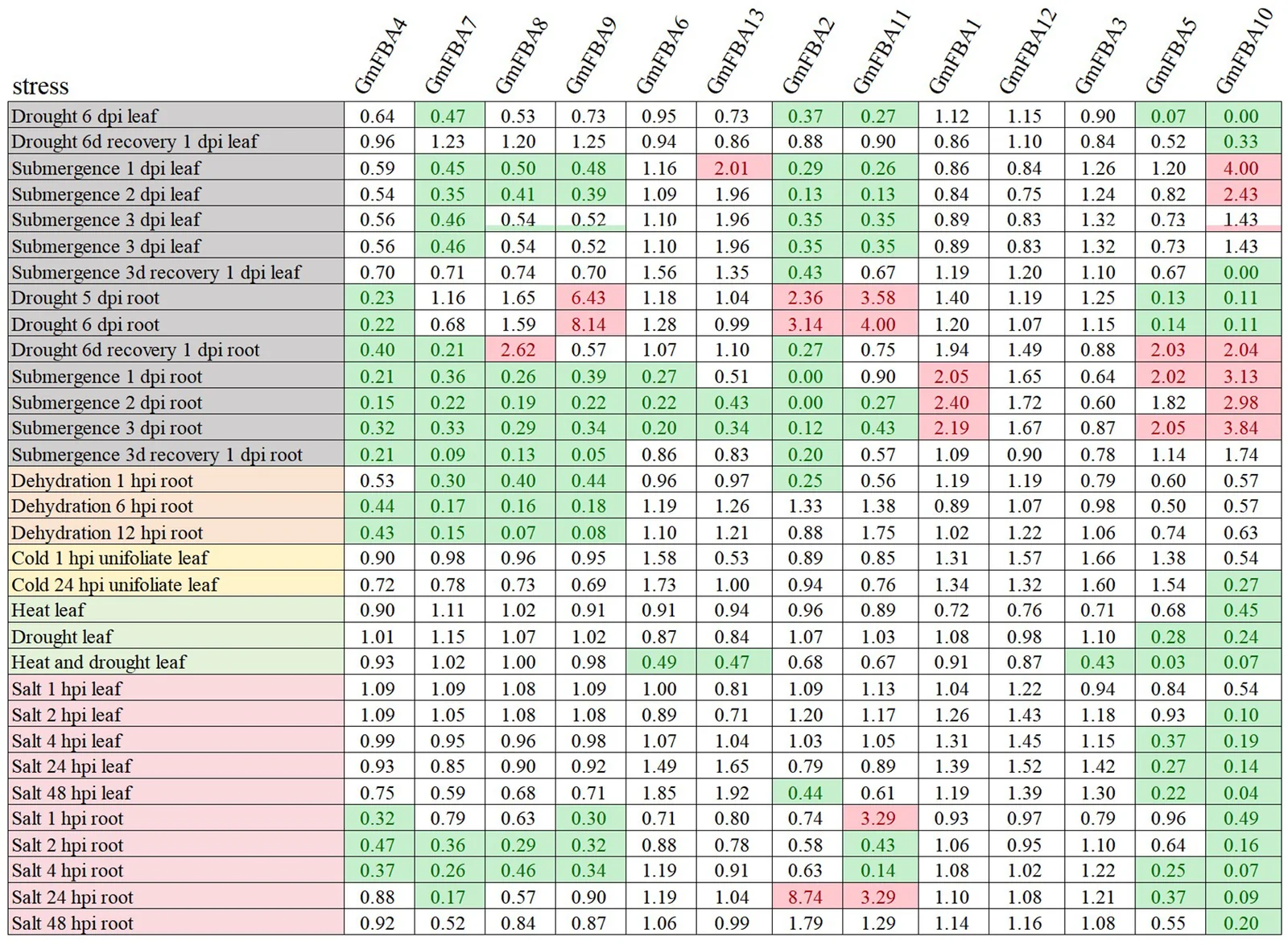
Expression level changes of *GmFBA* genes under abiotic stresses. Fold changes of expression level of *GmFBA* genes under multiple abiotic stresses, including drought, submergence, dehydration, cold, heat, drought and heat, and salt. The numbers in boxes represent the fold change of a gene under stress treatment relative to mock or control. Red and green boxes indicate upregulation (FC ≥ 2.0) and downregulation (FC ≤ 0.5), respectively.

To comprehensively characterize stress-responsive expression patterns of *GmFBA* genes, we analyzed transcriptomic datasets from multiple biotic and abiotic stress experiments (**Supplementary Table 4**). Differential expression thresholds were defined as ≥ 2-fold upregulation (fold change, FC ≥ 2.0) or ≤ 0.5-fold downregulation (FC ≤ 0.5) relative to untreated controls. Biotic stress analysis (**Figure 6**; **Supplementary Table 5**) revealed that most *GmFBA* genes maintained relatively stable expression across aphid infestation (FC 0.64-1.60) and *Phialophora gregata* infection (FC 0.56-1.59), and SMV infection (FC 0.73-1.28). Under *Aspergillus oryzae* and *Rhizopus oligosporus* infection, *GmFBA2* and *GmFBA11* showed transient upregulation at early time points (FC up to 4.00) followed by sharp downregulation at later stages (FC as low as 0.04-0.08). *GmFBA5* was consistently upregulated in roots following *Fusarium virgiforme* infection, and *GmFBA9* was strongly upregulated by cyst nematode in the TN09–29 line (FC 11.33). Under abiotic stresses (**Figure 7**; **Supplementary Table 5**), *GmFBA* genes exhibited more pronounced differential expression. Drought stress induced strong upregulation of *GmFBA8* and *GmFBA9* in roots (FC up to 8.14), while *GmFBA5* and *GmFBA10* were significantly suppressed (FC as low as 0.11). Under submergence stress, *GmFBA10* was significantly upregulated in leaves (FC 4.00). In roots, three *GmFBA* genes (*GmFBA1*, *GmFBA5*, and *GmFBA10*) showed moderate upregulation, with FC values ranging from 2.05 to 3.84. Salt stress induced gradual downregulation of *GmFBA5* and *GmFBA10* in leaves over time (FC as low as 0.04), while *GmFBA2* showed strong upregulation in roots at 24 h (FC 8.74). Drought and heat combined stress elicited moderate downregulation of most *GmFBA* genes, with *GmFBA5* and *GmFBA10* showing the most pronounced suppression (FC 0.03 and 0.07, respectively). Dehydration, cold, and heat stresses elicited minimal transcriptional changes, with all *GmFBA* genes retaining relatively stable expression (FC 0.44-1.73).

### Transcriptomic and qRT-PCR validation of *GmFBA* gene expression under salt-alkali stress

**Figure 8:**
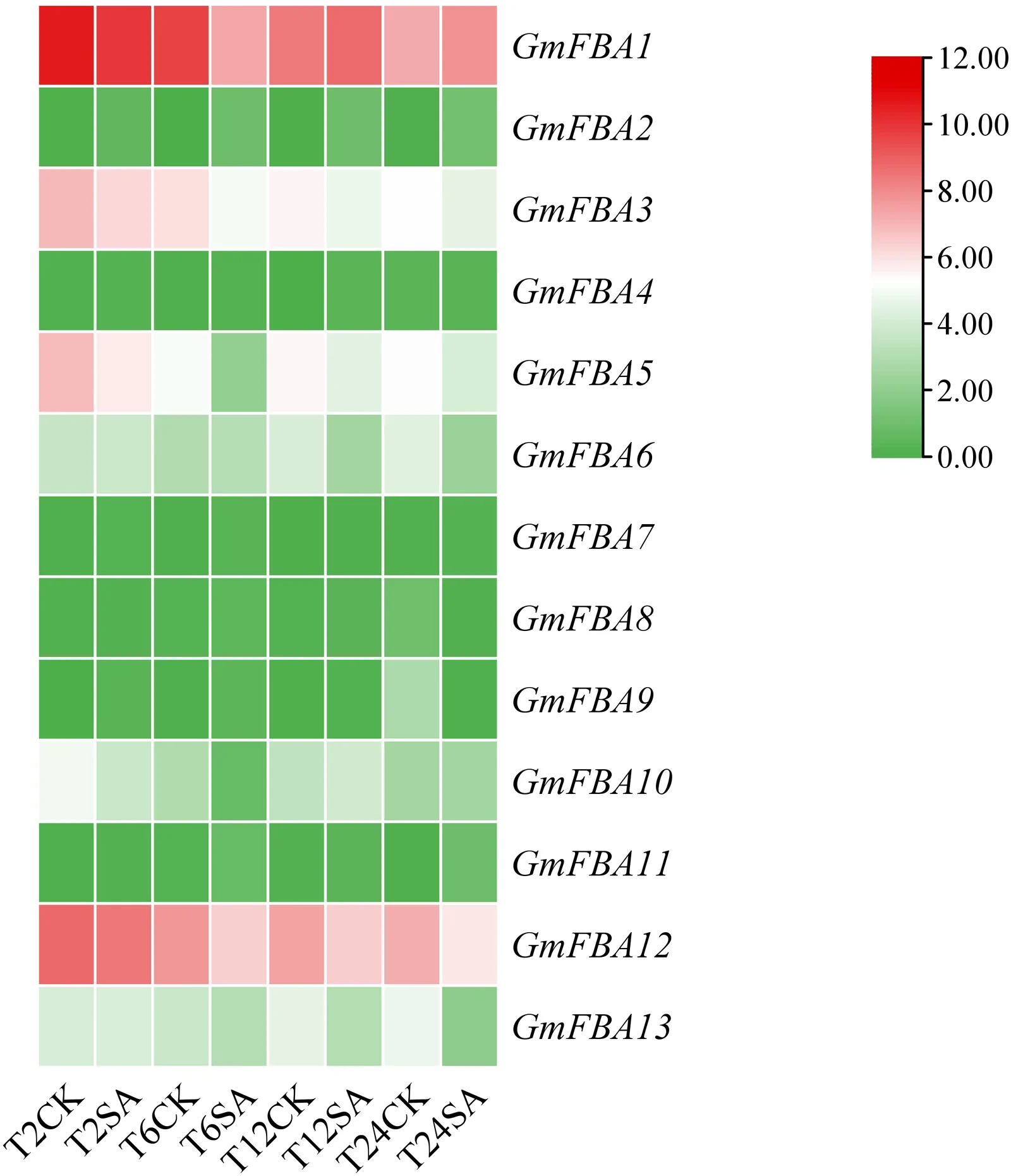
Heatmap of *GmFBA* gene expression profiles in soybean roots under salt-alkali stress. RNA-seq was performed on root samples collected at 2, 6, 12, and 24 hours after treatment with 150 mM Na^+^ (pH 8.9). The heatmap was generated using log_2_(FPKM + 1)-transformed values. Red and green boxes indicate upregulation (FC ≥ 2.0) and downregulation (FC ≤ 0.5), respectively.

**Figure 9:**
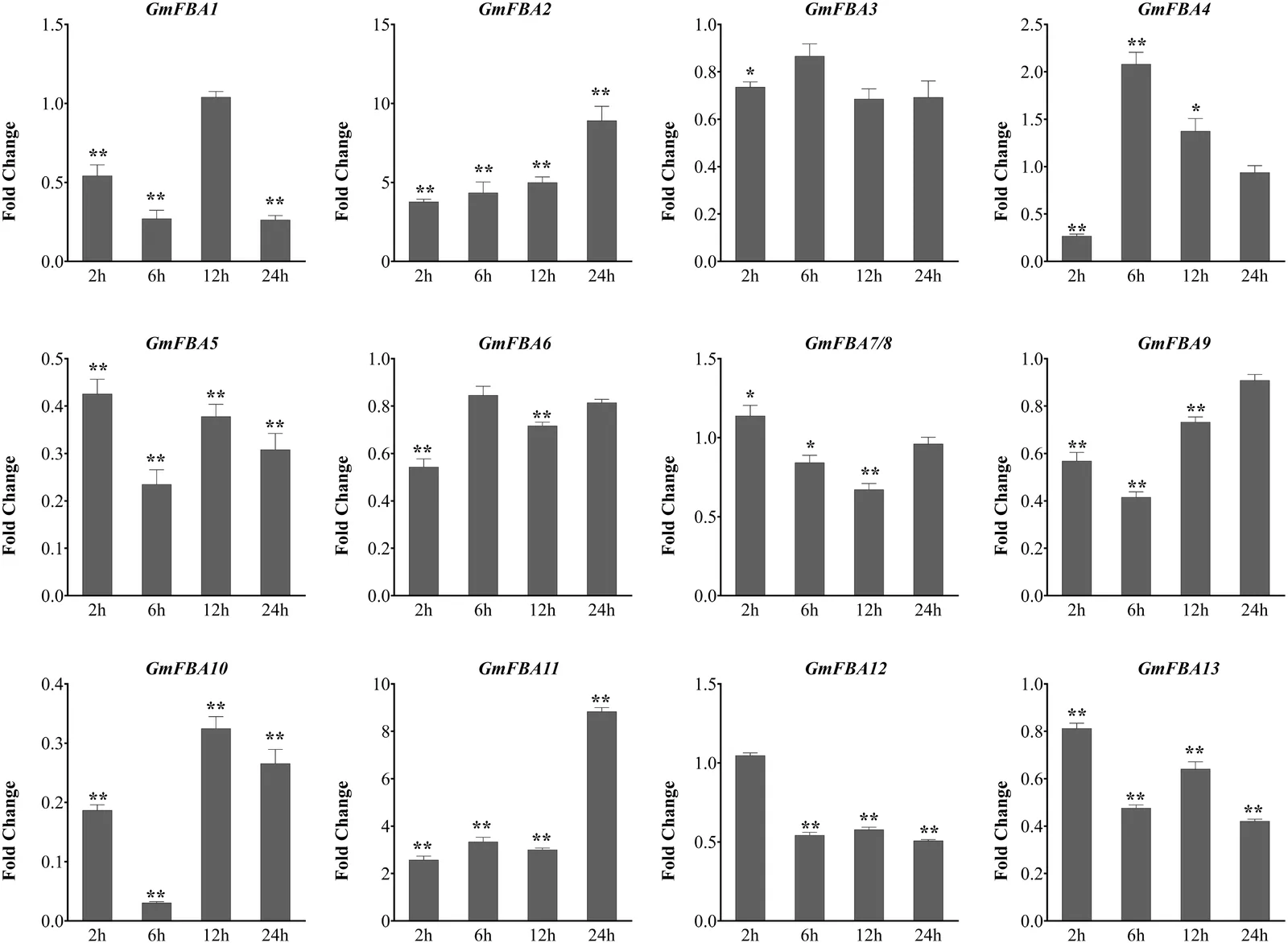
Expression levels of *GmFBA* genes under salt-alkali stress in soybean roots. qRT-PCR was performed on root-expressed *GmFBA* genes in triplicate (n = 3) at 2, 6, 12, and 24 hours after treatment with 150 mM Na^+^ (pH 8.9). The expression level of each gene at each time point was calculated relative to its corresponding control (CK) at the same time point, and each control was set to 1.0. Error bars indicate SE, and *p* < 0.05 (*) or *p* < 0.01 (**).

To further investigate the expression patterns of *GmFBA* genes under salt-alkali stress, we performed transcriptome sequencing of soybean roots treated with 150 mM Na^+^ (pH 8.9) at 2, 6, 12, and 24 h. Principal-component-analysis (PCA) confirmed high reproducibility of all transcriptome samples (**Supplementary Figure 2**). The FPKM values of all *GmFBA* genes under salt-alkali stress are provided in **Supplementary Table 6**. As shown in **Figure 8**, the 13 *GmFBA* genes exhibited distinct temporal expression patterns. *GmFBA2* and *GmFBA11* were consistently upregulated across all time points. In contrast, *GmFBA5* and *GmFBA10* were strongly downregulated, while the remaining genes showed relatively stable or moderate expression changes. To validate the transcriptomic results, we performed qRT-PCR analysis on the same root samples, Pearson-correlation analysis revealed a strong positive correlation (R = 0.9091, *p* < 0.001) between RNA-seq and qRT-PCR data (**Supplementary Figure 3**). The expression level of each gene at each time point was calculated relative to its corresponding control at the same time point. The 13 *GmFBA* genes fell into three distinct expression categories under salt-alkali stress (**Figure 9**). Exact *p* value for all comparisons are provided in **Supplementary Table 7**. Two genes, *GmFBA2* and *GmFBA11*, exhibited sustained and pronounced up-regulation across all time points, with progressively increasing expression levels reaching the highest induction at 24 h (FC 8.58 and 8.80, respectively). In contrast, four genes *GmFBA1*, *GmFBA5*, *GmFBA10*, and *GmFBA13* were strongly down-regulated, with *GmFBA10* showing the most drastic suppression (FC as low as 0.03 at 6 h). The remaining six genes (*GmFBA3*, *GmFBA4*, *GmFBA6*, *GmFBA7/8*, *GmFBA9*, and *GmFBA12*) showed only transient or moderate expression changes that did not consistently exceed the two-fold threshold across time points. These results suggest that *GmFBA2* and *GmFBA11* are promising candidate genes potentially involved in the response to salt-alkali stress.

## Discussion

FBAs are universally conserved enzyme positioned at the metabolic crossroads of glycolysis, gluconeogenesis, and the Calvin-Benson cycle (Berg et al., 2010; Fushinobu et al., 2011). In higher plants, *FBA* not only participates in cytosolic glycolytic energy production but also in plastidic carbon fixation, and its expression has been repeatedly shown to respond to salinity, drought, and extreme temperatures (Yamada et al., 2000; Fan et al., 2009; Khanna et al., 2014; Cai et al., 2018). Although the *FBA* gene family has been systematically characterized in model plants such as *Arabidopsis* (8 genes; Lu et al., 2012) and tomato (8 genes; Cai et al., 2016), its composition and evolution have remained largely unexplored in soybean. In this study, we identified 13 *GmFBA* genes in the soybean genome, which are unevenly distributed across nine of the 20 chromosomes (**Table 1**, **Figure 1**). The observed gene number is modest compared with that of polyploid species such as tobacco (16 genes; Zhao et al., 2021), cotton (17 genes; Li et al., 2021), wheat (21 genes; Lv et al., 2017), and rapeseed (22 genes; Zhao et al., 2019), consistent with the paleopolyploid origin of soybean. Synteny analysis identified nine segmental duplication pairs and one tandem duplication pair (*GmFBA7* and *GmFBA8*) (**Figure 1**), yet all duplicated pairs exhibited *K*a*/K*s ratios far below 1 (0.03-0.19; **Table 2**), indicating exceptionally strong purifying selection. Consequently, most non-synonymous mutations are likely deleterious and have been eliminated over evolutionary time (Loewe et al., 2006). This extreme conservation underscores the non-redundant and essential roles of *GmFBAs* in central carbon metabolism. Any alteration that perturbs their catalytic efficiency or subcellular compartmentation would likely disrupt the finely tuned balance between glycolysis and photosynthetic carbon assimilation, with potentially severe consequences for growth and stress adaptation. Hence, the strong purifying selection acting on *GmFBA* genes reflects an evolutionary strategy that maintains a stable and efficient core metabolic network, which is particularly critical for a crop like soybean that faces frequent environmental fluctuations.

Exon-intron architecture provides critical insights into the evolutionary and functional relationships within gene families (Xu et al., 2012). In diverse plant species, *FBA* genes exhibit distinct exon-intron organization patterns corresponding to their subcellular localization (Lu et al., 2012; Cai et al., 2016; Carrera et al., 2021). Our structural analysis revealed that all *GmFBA* genes adhere to this canonical configuration (**Figure 3**). Specifically, Groups I and II (chloroplast-localized) contain six exons and five introns with a longer N-terminal extension (34–44 amino acids) that likely functions as a chloroplast transit peptide, whereas Groups III and IV (cytoplasm-localized) exhibit a more compact structure consisting of only three exons and two introns. The additional introns in Groups I and II may harbor regulatory elements responsive to light or developmental signals, consistent with observations in *Arabidopsis* and other plants (Carrera et al., 2021; Uematsu et al., 2012). In contrast, the compact structures of Groups III and IV resemble typical cytosolic *FBA* genes in tomato and rice (Cai et al., 2016; Zhao et al., 2026), facilitating rapid transcription and processing to maintain basal glycolytic flux—an inference supported by the constitutive expression of Group IV genes (*GmFBA1*, *GmFBA3*, and *GmFBA12*) across all examined tissues (**Figure 5**).

In *Arabidopsis* and tomato, *FBA* gene family members exhibit tissue-specific expression dynamics, with certain isoforms showing hormone and environmental responsiveness (Lu et al., 2012; Cai et al., 2016; Gemperline et al., 2019). Promoter sequence analysis in this study revealed that the promoter regions of *GmFBA* genes contain multiple *cis*-acting elements associated with hormone responses (abscisic acid, methyl jasmonate, salicylic acid, gibberellin, and auxin) and stress responses (anaerobic induction, defense, wound, drought, and low temperature) (**Figure 4**). The widespread distribution of these elements suggests that the expression of *GmFBA* genes is finely regulated by diverse developmental and environmental signals. In soybean, our expression profiling revealed a hierarchical functional stratification among *GmFBA* genes. Group I genes were highly expressed in photosynthetic vegetative organs such as cotyledons, stems, and leaves, but not in roots (**Figure 5**), indicating their specific roles in photosynthetic tissues, probably participating in the Calvin cycle. Group II genes showed the highest expression in roots, suggesting that they may perform specialized metabolic functions in plastids of non-photosynthetic tissues, such as providing precursors for amino acid synthesis. Notably, *GmFBA5* and *GmFBA10* exhibited root-preferential expression (**Figure 5**), implying specialized functions in root development or metabolism.

Abiotic stresses are major environmental factors limiting agricultural productivity (Wang et al., 2020; Zhang et al., 2022). Substantial evidence has demonstrated that plant *FBA* genes are involved in responses and adaptation to salt, drought, and temperature stresses (Yamada et al., 2000; Fan et al., 2009; Khanna et al., 2014; Cai et al., 2018). For example, salt stress alters plastidic *FBA* expression in rice (Yamada et al., 2000); *FBA* transcription is induced by salt and drought in the halophyte *Sesuvium portulacastrum* (Fan et al., 2009); downregulation of *SlFBA7* reduces tolerance to low temperature stress in tomato (Cai et al., 2018). In this study, analysis of publicly available RNA-seq data revealed that *GmFBA* genes exhibited diverse expression patterns under multiple abiotic stresses (**Figure 7**). Among the identified genes, *GmFBA4*, *GmFBA7*, and *GmFBA13* exhibited upregulation under a majority of stress conditions, indicating their potential role as general stress-responsive metabolic regulators. Notably, drought and salt stress specifically induced the expression of *GmFBA8*, *GmFBA9* and *GmFBA2*, *GmFBA11* in the roots, respectively. This interplay of stress specificity and tissue specificity suggests that soybean roots selectively activate distinct members of the *FBA* gene family in response to varying types of stress, thereby precisely modulating carbon metabolic flux to accommodate the specific energy requirements associated with each stress condition. In addition to abiotic stresses, plant *FBA* genes are also involved in biotic stress responses. Adverse biotic stress conditions pose serious threats to plant growth and development (Li et al., 2024c; Xie et al., 2021; Lv et al., 2017). To comprehensively characterize the stress-responsive expression patterns of *GmFBA* genes, we analyzed publicly available RNA-seq data under multiple biotic stress conditions (**Figure 6**). During fungal infections, such as those caused by *Aspergillus oryzae* and *Rhizopus oligosporus*, *GmFBA2* demonstrated a transient upregulation in the early stages, followed by a marked downregulation in later stages. This distinct temporal expression pattern implies that plants may rapidly augment glycolytic flux to supply energy and metabolic substrates necessary for defense responses during the initial phase of infection. Conversely, *GmFBA5* is strongly induced in roots upon infection by *Fusarium virgiforme*, suggesting that this gene may play a specific role in conferring broad-spectrum disease resistance in soybean roots. In contrast, the expression levels of most *GmFBA* genes remain relatively stable under aphid, *Phialophora gregata*, and SMV infection, indicating that these biotic stresses may not depend on *FBA*-mediated carbon metabolic reprogramming. In summary, the *GmFBA* gene family exhibits clear functional divergence in response to biotic and abiotic stresses in soybean: certain members act as general regulators, broadly responding to multiple stresses, while others have evolved highly specific stress-responsive capabilities, enabling adaptation to various environmental challenges.

In natural environments, salt stress is primarily induced by neutral salts (NaCl and Na_2_SO_4_), whereas alkali stress is mainly caused by alkaline salts (NaHCO_3_ and Na_2_CO_3_), which lead to distinct physiological challenges (Zhang et al., 2023; Wu et al., 2025). Salt-alkali stress adversely affects crop growth, cellular membranes, hampers photosynthesis, generates harmful compounds, and hinders nutrient uptake, ultimately resulting in diminished crop growth and decreased productivity (Ali et al., 2012; Cao et al., 2019). At present, more than 900 million hectares, constituting approximately 7 percent of the global land area, are susceptible to salt-alkali. This presents significant threats to terrestrial ecosystems and agricultural productivity on a global scale (Haj-Amor et al., 2022; Sahab et al., 2021; Zhang et al., 2022; Zhang and Wu, 2023). To investigate the expression patterns of *GmFBA* genes under salt-alkali stress, we performed transcriptome sequencing of soybean roots at 2, 6, 12, and 24 h after treatment. The heatmap generated from log_2_(FPKM + 1)-transformed values revealed that *GmFBA2* and *GmFBA11* were consistently upregulated across all time points, while *GmFBA5* and *GmFBA10* were downregulated (**Figure 8**; **Supplementary Table 6**). To validate these transcriptomic findings, we further performed qRT-PCR analysis of 13 root-expressed *GmFBA* genes under the same conditions. The results revealed three distinct expression patterns among these genes (**Figure 9**). The qRT-PCR results were largely consistent with the transcriptome data in terms of overall expression trends, confirming the sustained up-regulation of *GmFBA2* and *GmFBA11*. Minor quantitative discrepancies in fold-change magnitudes were detected between the two platforms. These deviations mainly stem from their intrinsic differences in detection sensitivity and data-normalization workflows. RNA-seq quantifies the complete pool of transcripts at the genome-wide level, whereas qRT-PCR enables targeted amplification of selected transcripts and generally delivers a broader dynamic range and higher quantitative precision for individual target genes. The upregulation of *GmFBA2* and *GmFBA11* may enhance cytosolic glycolytic flux to provide ATP for ion transport and osmolyte biosynthesis, whereas the downregulation of most other members may reflect feedback inhibition or a strategic reallocation of carbon skeletons from growth-oriented anabolism toward stress defense metabolism. This divergent transcriptional response may reflect a potential strategic trade-off in energy and carbon management under salt-alkali conditions. FBAs act as a central enzyme in both glycolysis and the Calvin cycle, and its expression patterns are closely associated with plant salt-stress adaptation and carbon partitioning (Cai et al., 2016; Huang et al., 2021). The induced expression of *GmFBA2* and *GmFBA11* potentially sustains glycolytic ATP production, which may contribute to supporting ion transport and osmolyte biosynthesis during stress. Notably, *GmFBA2* is the ortholog of *Arabidopsis AtFBA2*, which participates in photosynthetic energy metabolism and starch synthesis within the Calvin cycle (Kao et al., 2025). In contrast, the concomitant downregulation of other family members, particularly *GmFBA10*, may help conserve cellular energy and partially redirect carbon skeletons from growth-oriented anabolism toward stress-defense metabolism. This pattern of selective activation alongside partial suppression likely represents a flexible regulatory strategy, in which plants possibly maintain essential glycolytic flux through specific *FBA* isoforms while reducing unnecessary energy consumption associated with broad family-wide expression. Such an aldolase-associated metabolic trade-off under energy-limited conditions has been previously reported in eukaryotic species (Zhai et al., 2025), implying that this regulatory mode may facilitate soybean root adaptation to saline-alkali environments by remodeling carbon allocation and energy utilization.

Our comprehensive genome-wide analysis of the *GmFBA* gene family, integrated with transcriptomic expression data, provides correlative evidence suggesting a link between the transcriptional variation of *GmFBA* members and the reallocation of carbon flow in soybean roots under salt-alkali stress conditions. While our findings offer initial insights into the potential involvement of specific *GmFBA* members in stress responses, predictions regarding their functional roles based solely on gene structural characteristics and transcript abundance remain inconclusive in establishing their causal roles in salt-alkali tolerance. Importantly, all expression validation experiments related to salt-alkali stress in this study were conducted using a single salt-alkali-tolerant soybean cultivar. Consequently, the stress-responsive expression patterns observed may be specific to this genotype and may not be applicable across diverse soybean germplasms. To better understand how *GmFBA2* and *GmFBA11* influence carbon metabolism, future research should examine different soybean cultivars with varying salt-alkali tolerance to confirm the pathway’s consistency across genetic backgrounds. This includes protein quantification, aldolase activity measurement, glycolytic intermediate profiling, and CRISPR-Cas9 knockout and overexpression studies to clarify the roles of *GmFBA2* and *GmFBA11* in growth-defense balance and root ion homeostasis under stress. These studies will enhance our understanding of FBA-mediated adaptation in soybeans.

## Conclusions

The study identified 13 *GmFBA* genes in the soybean genome, unevenly distributed across 9 of 20 chromosomes and grouped into five phylogenetic categories. Segmental duplication was the main driver of gene family expansion, with duplicated pairs under strong purifying selection (low *K*a/*K*s ratios of 0.03-0.19). Subcellular localization showed Group I and II proteins in the chloroplast and Groups III and IV in the cytoplasm, linked to an extended N-terminal transit peptide in the former. Expression analysis revealed Group I genes are highly expressed in photosynthetic organs, Group II in roots, and Group IV, consisting of three genes (*GmFBA1*, *GmFBA3*, and *GmFBA12*), was constitutively expressed across all tissues. Notably, under salt-alkali stress, *GmFBA2* and *GmFBA11* were consistently upregulated, suggesting a reprogramming of carbon metabolism. This study is the first to comprehensively characterize the *FBA* gene family in soybeans, providing a foundation for further research, particularly on the functional validation of candidate genes such as *GmFBA2* and *GmFBA11* for potential application in salt-alkali tolerance breeding.

## Data Availability

The datasets presented in this study can be found in online repositories. The names of the repository/repositories and accession number(s) can be found in the article/**Supplementary Material**.

## Supplementary material

Supplementary Figure 1 The sequences information of 5 conserved motifs identified in GmFBA proteins.

Supplementary Figure 2 Principal-component analysis (PCA) of all RNA-seq samples.

Supplementary Figure 3 Pearson correlation analysis of RNA-seq and qRT-PCR fold-change values. Correlation analysis was performed based on raw fold-change (FC) values derived from 44 gene-timepoint pairs (11 genes and four treatment time points). The homologous gene pair *GmFBA7/GmFBA8* was excluded due to the inability of qRT-PCR primers to distinguish their expression signals. A strong positive correlation was observed between RNA-seq and qRT-PCR results (R = 0.9091, *p* < 0.001), indicating high consistency between the two detection methods.
