# Supplementary material for: Genome-wide analysis of the FBA gene family in soybean and their expression profile under salt-alkali stress
Source: Front Plant Sci. 2026 Sep 10;17:1908014. doi: 10.3389/fpls.2026.1908014 (PMC13601244; doi:10.3389/fpls.2026.1908014)
Supplement: Supplementary file 1 [file DataSheet1.zip › Supplementary files/Supplementary Table S1;S2;S4.docx]

**Supplementary Table S1 The sequences of the primers in this study.**

| **Gene name** | **Forward primer** | **Reverse primer** | **purpose** |
| --- | --- | --- | --- |
| *Tubulin* | TCTTGGACAACGAAGCCATCT | TGGTGAGGGACGAAATGATCT | qRT-PCR |
| *GmFBA1* | GAATGTGAAGAAGGCTCAGGAAGC | CGATTATTCTGGGAAGAGTCAAACCAC | qRT-PCR |
| *GmFBA2* | TGGACTCTCTCATTCTCATTCGGG | ACTCTCAGAAGTCAAGCCACTTCC | qRT-PCR |
| *GmFBA3* | AGGAGGCAACCCTCAACC | GGAGGCACCCTCAGCAAG | qRT-PCR |
| *GmFBA4* | CAGTCTCCAAACCCATGGCAC | TTGACGAACATTCCCCGAGTG | qRT-PCR |
| *GmFBA5* | AGCAGAGCACGCTCAAGG | ACCAATCTCACACCTCTCTCTTAGTAC | qRT-PCR |
| *GmFBA6* | GCAAGGACACCCTGAGAATGT | TAAGTCCCCTTCGAATTCACTGCT | qRT-PCR |
| *GmFBA7/8* | CTGGTGGACAGTCTGAGGTTG | CCTTCTTGGCTTCCTCAGATTCAC | qRT-PCR |
| *GmFBA9* | CCTTCTTGGCTTCCTCAGATTCAC | CTGCTGGTGTTGCTGCCTAAC | qRT-PCR |
| *GmFBA10* | GGGTGGAAAGGACGAGAATATCCC | TACCACCTACGACCGAGAATATCC | qRT-PCR |
| *GmFBA11* | TGTCTGGTGGGCAAAGTGAAG | AACTCTCAGAAGTCAAGCCACTTC | qRT-PCR |
| *GmFBA12* | GCCATTAACCAGGTCAATGGAAAG | AATACCGATTCCTACCCGCAC | qRT-PCR |
| *GmFBA13* | GCCATGAACCAAAGTCCCAAC | AAACATTCCCTTCTTGGCCTCTTC | qRT-PCR |

**Supplementary Table S2 FPKM values of *GmFBA* genes in different organs and tissues of soybean**

| Name | Root | Cotyledon | Stem | Axillary bud | Leaf bud | Leaf | Flower | 3 wap pod | 4 wap pod | 5 wap pod | 3 wap seed | 5 wap seed | 6 wap seed | 8 wap seed |
| --- | --- | --- | --- | --- | --- | --- | --- | --- | --- | --- | --- | --- | --- | --- |
| *GmFBA4* | 0.79 | 957.78 | 335.10 | 117.02 | 419.98 | 1222.41 | 76.21 | 58.58 | 121.77 | 138.11 | 4.38 | 5.92 | 3.91 | 4.69 |
| *GmFBA7* | 0 | 610.47 | 135.19 | 35.52 | 133.68 | 940.31 | 14.60 | 21.53 | 34.34 | 65.49 | 6.41 | 31.64 | 22.99 | 27.79 |
| *GmFBA8* | 0.19 | 856.84 | 166.91 | 63.22 | 251.49 | 1378.57 | 29.65 | 43.23 | 89.34 | 166.40 | 28.54 | 55.06 | 34.88 | 30.98 |
| *GmFBA9* | 0.47 | 1543.57 | 394.73 | 146.58 | 556.63 | 2675.15 | 62.78 | 105.77 | 170.52 | 313.73 | 31.55 | 172.74 | 106.89 | 126.65 |
| *GmFBA6* | 56.46 | 12.19 | 29.95 | 33.03 | 18.14 | 12.02 | 37.86 | 36.72 | 33.99 | 24.18 | 34.91 | 28.53 | 13.50 | 7.76 |
| *GmFBA13* | 28.59 | 1.91 | 10.21 | 8.73 | 6.59 | 2.63 | 8.44 | 8.64 | 12.68 | 12.03 | 14.78 | 10.40 | 4.33 | 3.51 |
| *GmFBA2* | 0 | 89.64 | 9.02 | 1.78 | 27.79 | 150.96 | 0.48 | 0.92 | 0.67 | 1.13 | 0 | 0.07 | 0 | 0 |
| *GmFBA11* | 0 | 220.70 | 25.18 | 3.66 | 29.39 | 370.73 | 0.89 | 2.37 | 4.62 | 5.24 | 0.28 | 0.15 | 0.10 | 0.07 |
| *GmFBA1* | 284.47 | 60.34 | 235.65 | 240.73 | 208.02 | 79.99 | 160.85 | 281.93 | 358.72 | 245.35 | 286.03 | 437.00 | 335.92 | 255.02 |
| *GmFBA12* | 393.70 | 33.61 | 133.87 | 152.38 | 187.97 | 60.32 | 116.93 | 303.35 | 352.90 | 220.36 | 318.85 | 297.08 | 258.61 | 165.88 |
| *GmFBA3* | 100.36 | 22.55 | 44.35 | 28.54 | 20.42 | 20.50 | 42.46 | 37.81 | 96.85 | 141.38 | 40.29 | 43.99 | 40.15 | 17.88 |
| *GmFBA5* | 103.73 | 0.95 | 11.30 | 8.11 | 7.14 | 4.00 | 2.56 | 11.65 | 6.78 | 5.48 | 1.60 | 0.88 | 0.32 | 0.10 |
| *GmFBA10* | 34.79 | 0 | 8.78 | 0.56 | 0.97 | 0 | 2.09 | 11.24 | 7.27 | 13.17 | 0.59 | 0.24 | 0 | 0.07 |

Note: wap, weeks after pollination

**Supplementary Table S4 The detailed information of RNA-Seq data of soybean under multiple biotic and abiotic stress**

| **Stress** | **Treatment and Sampling** | **Project and Reference article** |
| --- | --- | --- |
| Aphid | Leaf samples from caged areas of three soybean varieties under both aphid-infested and mock-treated conditions were harvested at 24, 48, and 96 hours post infestation (hpi). | PRJNA594515  Yao et al., 2020 |
| P. gregata | Leaf, stem, and root tissues of a resistant soybean genotype (PI 437970, carrying the Rbs3 gene) under both P. gregata-infected and mock-treated conditions were harvested at 12, 24, and 36 hours post infection (hpi). | PRJNA548564  McCabe CE and Graham MA., 2020 |
| *Aspergillus oryzae* or  *Rhizopus oligosporus* | After 48 h when germination started, germinated soybean seeds were inoculated with 1% (w/w) of *A. oryzae* or *R. oligosporus* spore and then incubated under a humidity of 100%. Soybeans seedlings were sampled every 24 hours for 8 days from the start of germination. | PRJDB10183  Uchida et al., 2020 |
| Soybean mosaic virus | Two soybean cultivars were inoculated with SMV strain G7 (SMV-G7), and the inoculated unifoliolate leaves were collected at 0, 2, 4, 6, and 8 hours post inoculation (hpi). | PRJNA564957  DeMers et al., 2020 |
| *Fusarium virgiforme* | Soybean cultivar Sloan was inoculated with Fusarium virgiforme, and root tissues were collected at 0, 2, 4, 7, 10, and 14 days post inoculation (dpi), with three biological replicates per time point. | PRJNA549915  Baetsen et al., 2020 |
| Cyst nematode | Examining the changes in the transcriptome of two nearly isogenic lines (TN09-16 and TN09-29) under soybean cyst nematode infected and non-infected conditions. | PRJNA534069  Rambani et al., 2020 |
| Drought and submergence | Soybean was subjected to drought stress (5 d, 6 d) and submergence stress (1 d, 2 d, 3 d), with rewatering recovery (6 d drought + 1 d recovery) and reoxygenation recovery (3 d submergence + 1 d recovery), along with respective controls. Leaf and root tissues were collected separately for each treatment, with three biological replicates per treatment. | PRJNA574626  Tamang et al., 2021 |
| Dehydration | Soybean plants were removed from the germination paper and left in air under water-limiting conditions to impose dehydration stress, roots were harvested after 0, 1, 6 and 12 hour of stress treatment. | PRJNA246058  Belamkar et al., 2014 |
| Cold | Soybean seedlings were cold-treated (4℃), and the leaves were collected at 1 and 24 hours after treatment. | PRJNA482945  Yamasaki et al., 2016 |
| Heat and drought | Not given | PRJNA449574  No publication |
| Salt | The soybean plants were exposed to a 150 mM NaCl solution, roots were collected at 1, 2, 4, 24, 48 hours after treatment. | PRJNA432861  Wang et al., 2023 |

**References**

1.Yao, L.; Yang, B.; Ma, X.; Wang, S.; Guan, Z.; Wang, B.; et al. (2020). A genome-wide view of transcriptional responses during *Aphis glycines* infestation in soybean. *Int. J. Mol. Sci*. 21, 5191. doi:10.3390/ijms21155191

2.McCabe, C.E.; and Graham, M.A. (2020). New tools for characterizing early brown stem rot disease resistance signaling in soybean. *Plant Genome* 13, e20037. doi:10.1002/tpg2.20037

3.Uchida, K.; Sawada, Y.; Ochiai, K.; Sato, M.; Inaba, J.; and Hirai, M.Y. (2020). Identification of a unique type of isoflavone O-methyltransferase, *GmIOMT1*, based on multi-omics analysis of soybean under biotic stress. *Plant Cell Physiol.* 61, 1974-1985. doi:10.1093/pcp/pcaa112

4.DeMers, L.C.; Redekar, N.R.; Kachroo, A.; Tolin, S.A.; Li, S.; and Saghai Maroof, M.A. (2020). A transcriptional regulatory network of Rsv3-mediated extreme resistance against *Soybean mosaic virus*. *PLoS ONE* 15, e0231658. doi:10.1371/journal.pone.0231658

5.Baetsen-Young, A.; Man Wai, C.; VanBuren, R.; and Day, B. (2020). *Fusarium virguliforme* transcriptional plasticity is revealed by host colonization of maize versus soybean. *Plant Cell* 32, 336-351. doi:10.1105/tpc.19.00697

6.Rambani, A.; Pantalone, V.; Yang, S.; Rice, J.H.; Song, Q.;Mazarei, M.; et al. (2020). Identification of introduced and stably inherited DNA methylation variants in soybean associated with soybean cyst nematode parasitism. *New Phytol*. 227, 168-184. doi:10.1111/nph.16511

7.Tamang, B.G.; Li, S.; Rajasundaram, D.; Lamichhane, S.; and Fukao, T. (2021). Overlapping and stress-specific transcriptomic and hormonal responses to flooding and drought in soybean. *Plant J.* 107, 100-117. doi:10.1111/tpj.15276

8.Belamkar, V.; Weeks, N. T.; Bharti, A. K.; Farmer, A. D.; Graham, M. A.; and Cannon, S. B. (2014). Comprehensive characterization and RNA-Seq profiling of the HD-Zip transcription factor family in soybean (*Glycine max*) during dehydration and salt stress. *BMC Genomics* 15, 950. doi:10.1186/1471-2164-15-950

9.Yamasaki, Y.; and Randall, S. K. (2016). Functionality of soybean *CBF/DREB1* transcription factors. *Plant Sci.* 246, 80-90. doi:10.1016/j.plantsci.2016.02.007

10.Wang, M.; He, C.; Shi, G.; Yin, Q.; Zhang, H.; Yang, W.; et al. (2023). Genome-wide analysis of the *SCAMPs* gene family of soybean and functional identification of *GmSCAMP5* in salt tolerance. *BMC Plant Biol.* 23, 628. doi:10.1186/s12870-023-04649-2
